# *Rhus javanica* Gall Extract Inhibits the Differentiation of Bone Marrow-Derived Osteoclasts and Ovariectomy-Induced Bone Loss

**DOI:** 10.1155/2016/3284704

**Published:** 2016-05-30

**Authors:** Tae-Ho Kim, Eui Kyun Park, Man-Il Huh, Hong Kyun Kim, Shin-Yoon Kim, Sang-Han Lee

**Affiliations:** ^1^Biomedical Research Institute, Kyungpook National University Hospital, Daegu 41940, Republic of Korea; ^2^Skeletal Diseases Genome Research Center, Kyungpook National University Hospital, Daegu 41940, Republic of Korea; ^3^Department of Oral Pathology and Regenerative Medicine, School of Dentistry, IHBR, Kyungpook National University, Daegu 41940, Republic of Korea; ^4^Department of Ophthalmology, Graduate School of Medicine, Kyungpook National University, Daegu 41944, Republic of Korea; ^5^Department of Orthopedic Surgery, Graduate School of Medicine, Kyungpook National University, Daegu 41944, Republic of Korea; ^6^School of Food Science & Biotechnology, Kyungpook National University, Daegu 41566, Republic of Korea

## Abstract

Inhibition of osteoclast differentiation and bone resorption is a therapeutic strategy for the management of postmenopausal bone loss. This study investigated the effects of* Rhus javanica* (*R. javanica*) extracts on bone marrow cultures to develop agents from natural sources that may prevent osteoclastogenesis. Extracts of* R. javanica* (eGr) cocoons spun by* Rhus javanica* (Bell.) Baker inhibited the osteoclast differentiation and bone resorption. The effects of aqueous extract (aeGr) or 100% ethanolic extract (eeGr) on ovariectomy- (OVX-) induced bone loss were investigated by various biochemical assays. Furthermore, microcomputed tomography (*µ*CT) was performed to study bone remodeling. Oral administration of eGr (30 mg or 100 mg/kg/day for 6 weeks) augmented the inhibition of femoral bone mineral density (BMD), bone mineral content (BMC), and other factors involved in bone remodeling when compared to OVX controls. Additionally, eGr slightly decreased bone turnover markers that were increased by OVX. Therefore, it may be suggested that the protective effects of eGr could have originated from the suppression of OVX-induced increase in bone turnover. Collectively, the findings of this study indicate that eGr has potential to activate bone remodeling by inhibiting osteoclast differentiation and bone loss.

## 1. Introduction

Osteoporosis is a systemic bone disease characterized by reduction of bone mass, disruption of bone microarchitecture, and a consequent increase in bone fragility [1]. Generally, an imbalance in bone remodeling (an increase in bone resorption as compared to bone formation) leads to most of the adult skeletal diseases, including osteoporosis and rheumatoid arthritis [2]. This imbalance may be induced by several conditions, such as hormonal imbalance, chronic diseases, and medications, specifically corticosteroids [3]. Postmenopausal osteoporosis is the most common type of osteoporosis in women. It is linked to estrogen deficiency that results in overexpression of the proinflammatory cytokines, such as interleukin-1 (IL-1), IL-6, and tumor necrosis factor-*α* (TNF-*α*), within the bone microenvironment. It increases osteoclast activity that provokes bone resorption leading to a high bone turnover and bone loss [4–6].

Currently, hormone and antiresorptive therapies (e.g., bisphosphonates, selective tissue estrogenic activity regulators, and calcitonins) are generally used to treat osteoporosis [7, 8]. However, these pharmacological treatments have side effects. Particularly, the long-term use of estrogen after menopause increases the risks of breast, ovarian, and endometrial cancers besides blood clotting [9, 10]. Therefore, development of medicines from naturally occurring compounds with fewer side effects would greatly benefit postmenopausal women. Historically, several medicinal plants have been used to prevent and treat osteoporosis because they have fewer adverse reactions and are more suitable for long-term use than chemically synthesized medicines [11].

*Rhus chinensis* (*R. chinensis*, Anacardiaceae) is a tannin-rich herb widely used as a traditional Korean medicine for the treatment of diarrhea, prolonged coughing, and spontaneous perspiration [12].* R. javanica* (Bell.) Baker makes* R. javanica* cocoons while parasitizing on the leaf of* R. chinensis* undergoing the process of drying [13]. The constituents of eGr include syringic acid, gallic acid, methyl gallate (MG), protocatechuic acid, and 1,2,3,4,6-penta-O-galloyl-*β*-D-glucose [14, 15]. MG, a major component of* R. javanica*, exhibits strong antioxidant properties besides other biological activities such as antiviral, antitumor, and anti-inflammatory activities [16–18]. Recently, gallotannins from* R. javanica* were found to have inhibitory effects on adipocyte differentiation of 3T3-L1 cells [15]. Adipocytes and osteoblasts originate from bone marrow stromal cells (BMSCs) and are closely related to each other. Therefore, a balanced osteoblast and adipocyte differentiation is critical for the maintenance of healthy bones [19].

The bone protective effects of* R. javanica* in osteoporosis have not been evaluated. Therefore, the effects of* R. javanica* extracts (eGrs) on receptor activator of nuclear factor-*κ*B ligand- (RANKL-) mediated osteoclast differentiation were investigated* in vitro*. In addition, the efficacy of eGrs in suppressing OVX-induced bone loss was evaluated in mice.

## 2. Materials and Methods

### 2.1. Chemicals and Reagents

RANKL and macrophage colony-stimulating factor (M-CSF) were purchased from R&D Systems (Minneapolis, MN, USA). CellTiter 96 AQueous One Solution Cell Proliferation Assay kit was purchased from Promega (Madison, WI, USA). Tartrate-resistant acid phosphatase (TRAP; acid phosphatase kit 387-A) staining kit and dimethyl sulfoxide (DMSO) were obtained from Sigma Chemical Co. (St. Louis, MO, USA). All other chemicals and reagents were of analytical grade.

### 2.2. Preparation of eGr

Dried* R. javanica* was obtained from a medicinal shop (Yak-Jun Street, Daegu, Korea). The extraction of* R. javanica* was performed according to a published method [12]. To prepare the eGr, dried* R. javanica* was ground and extracted with distilled water (DW) (1 : 15 w/v, 60°C) or 100% ethanol (1 : 10 w/v, 45°C) for 24 h. After extraction, the solutions were filtered using a filter paper (number 4, Whatman, Buckinghamshire, UK), and the filtrates were freeze-dried in a freeze dryer. The freeze-dried powders obtained from aqueous extract (aeGr) or 100% ethanol (eeGr) were dissolved in DW or DMSO, respectively, and diluted with phosphate-buffered saline (PBS) for* in vitro* and* in vivo* assays. All voucher specimens (2012-RC) were deposited in the Laboratory of Food Enzyme Biotechnology, Kyungpook National University.

### 2.3. Osteoclast Differentiation

Osteoclast differentiation was induced as previously described [20]. Bone marrow cells extracted from the femora and tibiae of 6- to 8-week-old C57BL/6 mice (Daehan Biolink, Chungbuk, Korea) were cultured in *α*-minimal essential medium (*α*-MEM) supplemented with 10% fetal bovine serum (FBS) to generate osteoclasts. After 24 h of culture, the nonadherent cells were collected, centrifuged in Ficoll-Hypaque (Sigma Aldrich, St. Louis, MO) density gradient, and incubated in *α*-MEM containing 10% FBS and 10 ng/mL M-CSF for 3 days. Attached cells were considered bone marrow macrophages (BMMs). For the osteoclastogenesis experiments, BMMs were plated into a 96-well plate at a density of 3 × 10^4^ cells/well in *α*-MEM supplemented with 10% FBS, RANKL (20 ng/mL), and M-CSF (10 ng/mL), in the presence or absence of eGr.

### 2.4. TRAP Staining Assay

Osteoclast formation was evaluated by TRAP staining as previously described [21]. Briefly, the cells were fixed in 10% formaldehyde for 10 min and stained for TRAP with naphthol-AS-MX-phosphate and tartrate solution. TRAP-positive multinucleated cells (MNCs) containing ≥3 nuclei were scored. For measuring TRAP activity, fixed cells were incubated with 100 *μ*L substrate solution (3.7 mM* p*-nitrophenyl phosphate and 10 mM sodium tartrate in 50 mM citrate buffer, pH 4.6) at 37°C for 10 min. Subsequently, the reaction mixtures were transferred to new plates containing an equal volume of 0.1 N NaOH. Plates were read at 405 nm with a VERSAmax microplate reader (Molecular Devices, Sunnyvale, CA).

### 2.5. Cell Viability Assay

Cell viability was determined using CellTiter 96 AQueous One Solution Cell Proliferation Assay kit (Promega, Madison, WI). Mouse BMMs were incubated with several concentrations of the sample in the presence or absence of M-CSF (10 ng/mL) for 2 days. Subsequently, 20 *μ*L of CellTiter solution reagent was added to each well and the mixture was incubated for 2 h at 37°C. Plates were read at 490 nm using a 96-well microplate reader (Molecular Devices, Sunnyvale, CA).

### 2.6. Experimental Animals and Treatments

Animal experiments were performed in accordance with the principles and procedures approved by Kyungpook National University (KNU2012-63). Sixteen female C57BL/6 mice (8-week-old) were purchased from Daehan Biolink (Seoul, Korea) and acclimatized for 7 days prior to experiments. The mice were randomly divided into 4 groups (*n* = 5). The controls included sham-operated (sham) and OVX control (OVX_veh), which received equal volumes of phosphate-buffered saline (PBS) through oral gavage every day. The OVX treatment groups received 100 mg/kg body weight/day aeGr (OVX_aeGr) or eeGr (OVX_eeGr) via oral gavage for 6 weeks. At the end of treatment, all mice were euthanized by pentobarbital overdose. The bilateral femurs were excised, fixed with 3.7% formaldehyde (in PBS, pH 7.4) for 16 h, and subsequently stored (4°C) in 80% ethanol for *μ*CT.

### 2.7. Microcomputed Tomography (*μ*CT)

The fixed femurs were scanned using a SkyScan 1272 high-resolution *μ*CT system (Bruker, Kontich, Belgium). The X-ray tube voltage was 60 kV; the current was 166 *μ*A with a 0.25 mm thick Al filter. Exposure time was 424 ms and the image pixel size was 10 *μ*m. The acquired *μ*CT images were analyzed using the Comprehensive TeX Archive Network (CTAN) topographic reconstruction software (Bruker, Kontich, Belgium) to evaluate the bone parameters. For the bone analysis, a 0.7–2.3 mm region in the acquired images was selected as the region of interest (ROI) and the image information was obtained based on the automatic domain values yielded by the computer. The BMD of trabecular bone, bone volume (BV), bone volume density (the bone volume to tissue volume ratio, BV/TV), trabecular number (Tb.N), and trabecular separation (Tb.Sp) were measured and calculated.

### 2.8. Statistical Analysis

Statistical analyses were performed by two-tailed Student's *t*-test or one-way analysis of variance (ANOVA). Tukey's multiple comparison post hoc test was used to confirm significant differences between groups. A *p* value of less than 0.05 was considered significant.

## 3. Results

### 3.1. eGr Inhibits RANKL-Induced Osteoclastogenesis

**Figure 1:**
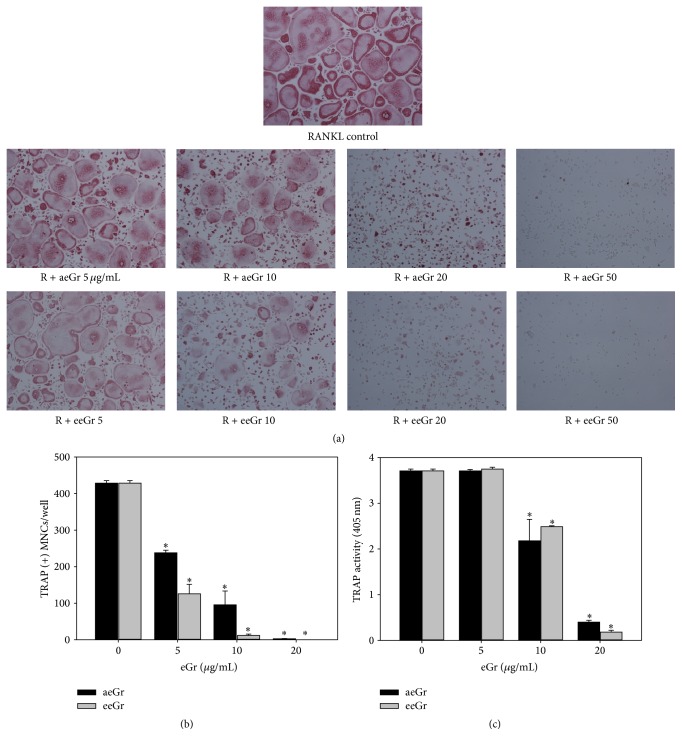
The effects of eGrs on the RANKL-induced osteoclast differentiation. (a) BMMs were cultured for 4-5 days in the presence of M-CSF (10 ng/mL), RANKL (20 ng/mL), and various concentrations of eGr. Osteoclasts were stained with TRAP. (b) The TRAP-positive MNCs containing three or more nuclei were scored as osteoclasts. (c) TRAP activity was measured at 405 nm. Results are represented as means ± SD of three independent experiments. ^*∗*^ *p* < 0.05, significant differences from the control.

**Figure 2:**
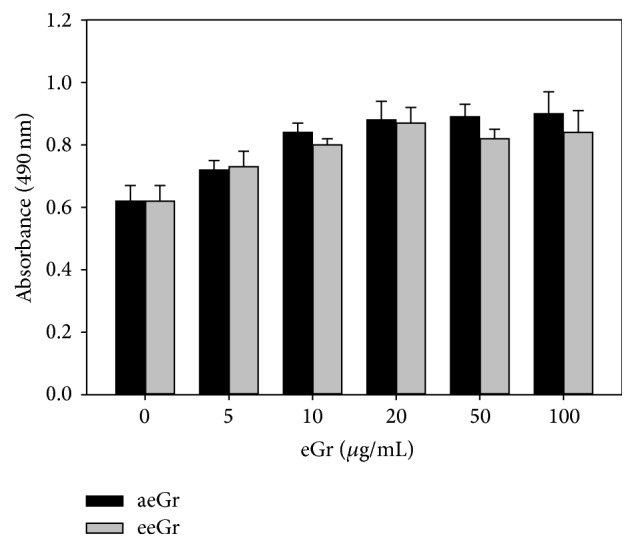
The effects of eGr on cell viability of BMMs measured by CellTiter 96® AQueous One Solution Cell Proliferation Assay kit. Mouse BMM cells were cultured in the presence of M-CSF (10 ng/mL) and various concentrations of eGr for 2 days. Data are represented as means ± SD.

BMMs, stimulated with RANKL (20 ng/mL) and M-CSF (10 ng/mL), were treated with various concentrations of eGr (1 *μ*g/mL to 50 *μ*g/mL) to examine the effects on osteoclast differentiation. The formation of osteoclast-like cells (TRAP-positive MNCs) and TRAP activity were analyzed. TRAP-positive MNCs were produced in the positive control (Figure 1(a), RANKL control) after 4 days of culture. However, the treatment with aeGr and eeGr considerably reduced the formation of MNCs in a dose-dependent manner as compared to the control. Both types of eGrs decreased osteoclast formation by more than 50.7% at 5 *μ*g/mL and by 95% at 20 *μ*g/mL (*p* < 0.05, Figure 1(b)). Besides inhibiting MNC formation, eGrs also inhibited the TRAP activity by more than 95% at 20 *μ*g/mL (Figure 1(c)). These results demonstrated that eGr effectively suppresses the number of TRAP-positive MNCs and the activity of TRAP. These inhibitory effects of eGr could be due to cytotoxicity or reduced growth of the osteoclast precursors. To ascertain that the inhibitory effects of eGr on the osteoclast formation were not due to its cytotoxicity, its effect on cell viability was evaluated. As shown in Figure 2, eGr was nontoxic and did not reduce the growth of BMM cells at the tested concentrations (up to 100 *μ*g/mL).

### 3.2. eGr Reduces OVX-Induced Bone Loss

**Figure 3:**
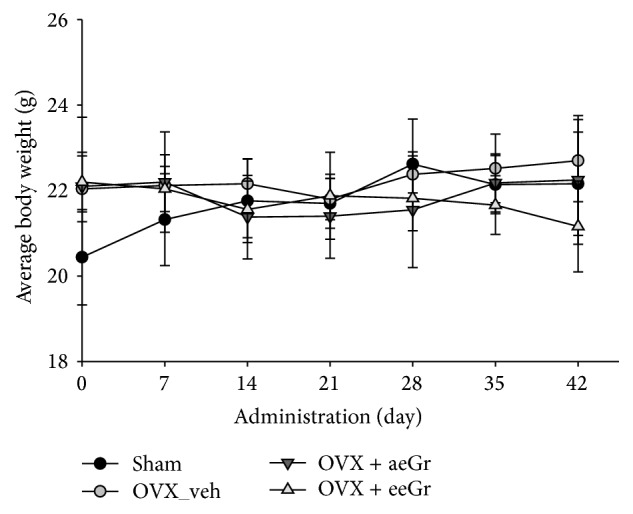
Changes in body weight of mice during treatment. The body weight was monitored daily in vehicle-treated sham-operated (black circle), vehicle-treated OVX (grey circle), aeGr-treated OVX (dark grey triangle), and eeGr-treated OVX (light grey triangle) mice during 6 weeks of treatment. Data are represented as means ± SD.

The eGr treatment suppressed RANKL-induced osteoclastogenesis* in vitro*. Therefore, it was investigated whether eGr would also exhibit the inhibitory effects* in vivo*. The OVX mice were given eGr (aeGr and eeGr) to determine its effect on bone loss* in vivo*.  Figure 3 shows the changes in body weight (BW) over the 7 weeks of experiment. The OVX procedure induced a higher weight gain than that of sham-operated controls (Figure 3; 0 d means 7 d after OVX). At the end of the 7 weeks of treatment, the mean body weight of the OVX group was slightly higher than that of the other groups. Particularly, the body weight of the eeGr-treated group (21.16 ± 1.06) was almost 7% lower than that of the vehicle-treated OVX group (22.70 ± 0.96). However, the differences between the groups did not reach statistical significance (Figure 3).

**Figure 4:**
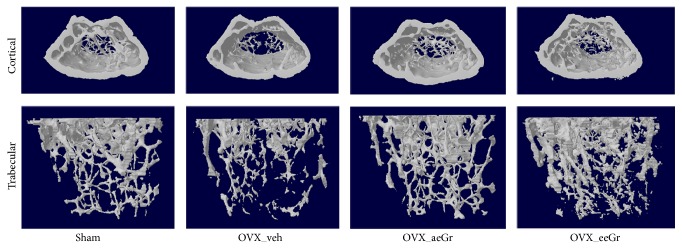
The effects of eGr treatment (100 mg/kg body weight/day) on the bone microarchitecture of the femur in OVX mice. Representative *μ*CT images of the distal femurs in each group are shown.

**Figure 5:**
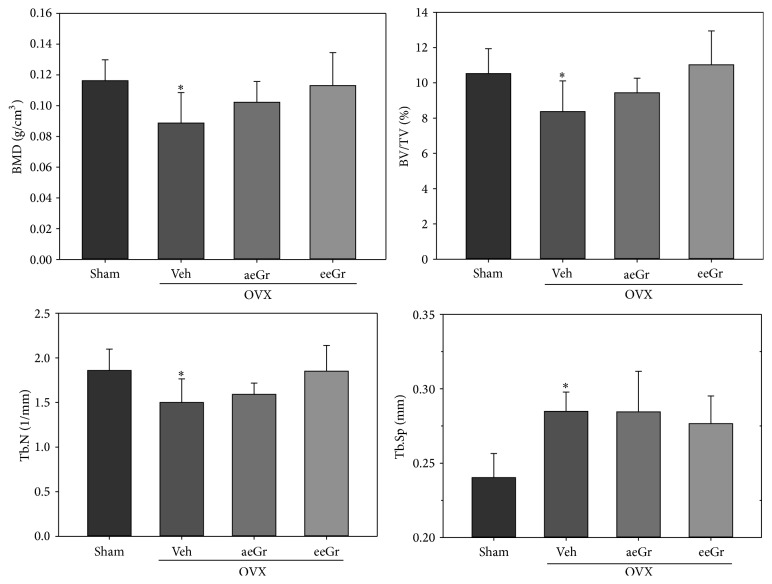
The effects of eGr treatment on the bone microarchitecture of the femur in OVX mice. The trabecular microarchitecture parameters (BV/TV, Tb.N, Tb.Sp, and trabecular BMD) of the femur (distal end) are shown. Data are represented as means ± SD. ^*∗*^ *p* < 0.05, significant differences from the sham control.

The OVX-induced bone loss was analyzed to determine the efficacy of eGr* in vivo*.  Figure 4 shows the representative *μ*CT images of the distal femur metaphyses from each group. As shown in the different dimension views, OVX apparently decreased trabecular bone in the femurs compared to the sham. The eGr treatment further reduced the trabecular bone in the femurs, which was higher than the OVX-induced decrease (Figure 4). In agreement with the *μ*CT images, treatment of OVX mice with eeGr significantly improved trabecular BMD by 27%, BV/TV by 32%, and Tb.N by 23% and decreased Tb.Sp by 3%, compared to the OVX_veh group. However, the differences did not reach statistical significance (Figure 5).

## 4. Discussion

*R. javanica* has long been recognized as a traditional Korean medicine for the treatment of diarrhea, prolonged coughing, and spontaneous perspiration as described in* Korean Dongui Bogam*. It contains several tannin-derived components, such as MG and gallic acid [15]. Tannic acid and its derivatives obtained from* R. javanica* have beneficial biological properties, including antioxidant, antiviral, antitumor, and anti-inflammatory activities [16]. However, their effect on bone metabolism has not been evaluated previously. The purpose of this study was to examine the effects of eGr on osteoclast differentiation* in vitro* and on the postmenopausal bone loss in OVX mice model. It was found that eGr(s) considerably suppressed the RANKL-induced osteoclast differentiation of BMM cells, as shown by the TRAP activity and multinucleated cell formation (Figures 1(a), 1(b), and 1(c)). Furthermore, eGr (at doses as high as 200 *μ*g/mL) did not show any cytotoxic response in mouse BMMs (Figure 2). These results suggest that the inhibitory effect of eGr on the osteoclast differentiation is not caused by cytotoxicity and that eGr has a selective effect on osteoclastogenesis.

There is a direct link between the lack of estrogen after menopause and the development of osteoporosis. During estrogen deficiency, the osteoclasts remove more bone than that formed by osteoblasts [1], resulting in bone loss. OVX animals have been widely used to study preventive treatments for postmenopausal osteoporosis. In these animals, the bone loss induced by removal of ovaries mainly results from the trabecular bone loss [22], like in postmenopausal women. The mechanical strength of a bone depends on the bone mass and bone quality and partly on the microstructure of trabecular bone [23]. As anticipated, OVX substantially reduced the bone parameters, including BMD, in the distal femur compared to the sham group. However, administration of eGr improved BMD of the distal femur in OVX mice (Figures 4 and 5). Furthermore, as shown in Figure 5, eGr also showed a significant effect on the trabecular bone microarchitecture, including Tb.N and Tb.Sp of the distal femur. Administration of eGr also improved BV/TV (+32%) and Tb.N (+23%) in the distal femur. These results demonstrated that eGr attenuates estrogen-dependent bone loss.

Moreover, estrogen deficiency induces an unregulated chronic inflammatory process by increasing the production of various osteoclastogenic cytokines, including TNF-*α*, IL-1, and IL-6, within the bone microenvironment [24].* R. javanica* and its constituents such as ethyl gallate and MG possess anti-inflammatory properties [25, 26]. MG, a major component of* R. javanica*, displays several biological activities, such as protection against DNA damage induced by oxidative stress [18] and antiapoptotic activity [17]. Furthermore, it inhibits the LPS-induced production of nitrogen oxide (NO) and IL-6. Oxidative stress contributes to the development of osteoporosis besides inflammation. Additionally, eGr shows strong and dose-dependent antioxidant activities, such as removal of reactive oxygen species (ROS) [12, 27]. Therefore, the inhibitory effect of eGr on bone loss may be associated with its anti-inflammatory and antioxidant activities.

In summary, eGr inhibits the osteoclast differentiation of BMMs and attenuates the OVX-induced bone loss* in vivo*. Although further studies will be required to provide further evidence, the findings of this study suggest that* R. javanica* may be a beneficial natural resource for prevention or treatment of bone loss induced by estrogen deficiency.

## References

[1] McNamara L. M. (2010). Perspective on post-menopausal osteoporosis: establishing an interdisciplinary understanding of the sequence of events from the molecular level to whole bone fractures. *Journal of the Royal Society Interface*.

[2] Boyle W. J., Simonet W. S., Lacey D. L. (2003). Osteoclast differentiation and activation. *Nature*.

[3] Goltzman D. (2002). Discoveries, drugs and skeletal disorders. *Nature Reviews Drug Discovery*.

[4] Kelly P. J. (1996). Is osteoporosis a genetically determined disease?. *British Journal of Obstetics and Gynaecology*.

[5] Riggs B. L., Khosla S., Melton L. J. (2002). Sex steroids and the construction and conservation of the adult skeleton. *Endocrine Reviews*.

[6] Pacifi R. (1998). Editorial: cytokines, estrogen, and postmenopausal osteoporosis—the second decade. *Endocrinology*.

[7] Weitzmann M. N., Pacifici R. (2006). Estrogen deficiency and bone loss: an inflammatory tale. *Journal of Clinical Investigation*.

[8] Rodan G. A., Martin T. J. (2000). Therapeutic approaches to bone diseases. *Science*.

[9] Reid I. R. (2002). Pharmacotherapy of osteoporosis in postmenopausal women: focus on safety. *Expert Opinion on Drug Safety*.

[10] Yeh I.-T. (2007). Postmenopausal hormone replacement therapy: endometrial and breast effects. *Advances in Anatomic Pathology*.

[11] Jia M., Nie Y., Cao D.-P. (2012). Potential antiosteoporotic agents from plants: a comprehensive review. *Evidence-Based Complementary and Alternative Medicine*.

[12] Lee S. H., Lee S. H. (2013). Comparison of antioxidant activity and *α*-glucosidase inhibiting activity by extracts of *Rhus javanica*. *Current Research on Agriculture and Life Sciences*.

[13] Choi J. G., Mun S. H., Chahar H. S. (2014). Methyl gallate from *Rhus javanica* successfully controls clinical isolates of *Salmonella* infection in both *in vitro* and *in vivo* systems. *PLoS ONE*.

[14] An R.-B., Oh H., Kim Y.-C. (2005). Phenolic constituents of Galla Rhois with hepatoprotective effects on tacrine- and nitrofurantoin-induced cytotoxicity in Hep G2 cells. *Biological and Pharmaceutical Bulletin*.

[15] Kwon O. J., Bae J.-S., Lee H. Y. (2013). Pancreatic lipase inhibitory gallotannins from galla rhois with inhibitory effects on adipocyte differentiation in 3T3-L1 cells. *Molecules*.

[16] Okuda T., Yoshida T., Hatano T. (1995). Hydrolyzable tannins and related polyphenols. *Fortschritte der Chemie Oganischer Naturstoffe*.

[17] Sciuto A. M., Moran T. S. (2001). Effect of dietary treatment with n-propyl gallate or vitamin E on the survival of mice exposed to phosgene. *Journal of Applied Toxicology*.

[18] Hsieh T.-J., Liu T.-Z., Chia Y.-C. (2004). Protective effect of methyl gallate from *Toona sinensis* (Meliaceae) against hydrogen peroxide-induced oxidative stress and DNA damage in MDCK cells. *Food and Chemical Toxicology*.

[19] Granéli C., Thorfve A., Ruetschi U. (2014). Novel markers of osteogenic and adipogenic differentiation of human bone marrow stromal cells identified using a quantitative proteomics approach. *Stem Cell Research*.

[20] Byung G. H., Hong J. M., Park J.-Y. (2008). Proteomic profile of osteoclast membrane proteins: identification of Na^+^/H^+^ exchanger domain containing 2 and its role in osteoclast fusion. *Proteomics*.

[21] Kim Y. W. O., Baek S.-H., Lee S.-H., Kim T.-H., Kim S.-Y. (2014). Fucoidan, a sulfated polysaccharide, inhibits osteoclast differentiation and function by modulating RANKL signaling. *International Journal of Molecular Sciences*.

[22] Kalu D. N. (1991). The ovariectomized rat model of postmenopausal bone loss. *Bone and Mineral*.

[23] Kinney J. H., Haupt D. L., Balooch M., Ladd A. J. C., Ryaby J. T., Lane N. E. (2000). Three-dimensional morphometry of the L6 vertebra in the ovariectomized rat model of osteoporosis: biomechanical implications. *Journal of Bone and Mineral Research*.

[24] Notoya M., Arai R., Katafuchi T., Minamino N., Hagiwara H. (2007). A novel member of the calcitonin gene-related peptide family, calcitonin receptor-stimulating peptide, inhibits the formation and activity of osteoclasts. *European Journal of Pharmacology*.

[25] Chae H.-S., Kang O.-H., Choi J.-G. (2010). Methyl gallate inhibits the production of interleukin-6 and nitric oxide via down-regulation of extracellular-signal regulated protein kinase in RAW 264.7 cells. *American Journal of Chinese Medicine*.

[26] Park P.-H., Hur J., Kim Y.-C., An R.-B., Sohn D. H. (2011). Involvement of heme oxygenase-1 induction in inhibitory effect of ethyl gallate isolated from *Galla Rhois* on nitric oxide production in RAW 264.7 macrophages. *Archives of Pharmacal Research*.

[27] Lee K., Kim J., Lee B. J. (2012). Protective effects of *Rhus javanica*, the excrescence produced by the sumac aphid, *Schlechtendalia chinensis*, on transient focal cerebral ischemia in the rat. *Journal of Insect Science*.

